# Effect of PET-CT misalignment on the quantitative accuracy of cardiac ^15^O-water PET

**DOI:** 10.1007/s12350-020-02408-6

**Published:** 2020-11-04

**Authors:** Jonny Nordström, Hendrik J. Harms, Tanja Kero, Maryam Ebrahimi, Jens Sörensen, Mark Lubberink

**Affiliations:** 1 Department of Surgical Sciences/Nuclear Medicine & PET, Uppsala University, Uppsala, Sweden; 2 Medical Physics, Uppsala University Hospital, Uppsala, Sweden; 3 Nuclear Medicine & Clinical Physiology, Aarhus University Hospital, Aarhus, Denmark; 4 Medical Imaging Centre, Uppsala University Hospital, Uppsala, Sweden; 5 Centre for Research and Development, Uppsala/Gävleborg County, Gävle, Sweden; 6 MedTrace Pharma A/S, Lyngby, Denmark; 7 PET Centre, Uppsala University Hospital, 751 85 Uppsala, Sweden

**Keywords:** PET, myocardial blood flow, image analysis, perfusion agents

## Abstract

**Background:**

Quantification of myocardial blood flow (MBF) with PET requires accurate attenuation correction, which is performed using a separate CT. Misalignment between PET and CT scans has been reported to be a common problem. The purpose of the present study was to assess the effect of PET CT misalignment on the quantitative accuracy of cardiac ^15^O-water PET.

**Methods:**

Ten clinical patients referred for evaluation of ischemia and assessment of MBF with ^15^O-water were included in the study. Eleven different misalignments between PET and CT were induced in 6 different directions with 10 and 20 mm amplitudes: caudal (+*Z*), cranial (− *Z*), lateral (±*X*), anterior (+*Y*), and anterior combined with cranial (+ *Y* and − *Z*). Blood flow was quantified from rates of washout (MBF) and uptake (transmural MBF, MBFt) for the whole left ventricle and the three coronary territories. The results from all misalignments were compared to the original scan without misalignment.

**Results:**

MBF was only minorly affected by misalignments, but larger effects were seen in MBFt. On the global level, average absolute deviation across all misalignments for MBF was 1.7% ± 1.4% and for MBFt 5.4% ± 3.2 Largest deviation for MBF was − 4.8% ± 5.8% (LCX, *X* + 20) and for MBFt − 19.3% ± 9.6% (LCX, *X* + 20). In general, larger effects were seen in LAD and LCX compared to in RCA.

**Conclusion:**

The quantitative accuracy of MBF from ^15^O-water PET, based on the washout of the tracer, is only to a minor extent affected by misalignment between PET and CT.

**Electronic supplementary material:**

The online version of this article (10.1007/s12350-020-02408-6) contains supplementary material, which is available to authorized users.

## Introduction

Cardiac positron emission tomography (PET) is increasingly being used in the clinical evaluation of coronary artery disease.^1^ Quantification of myocardial blood flow (MBF) has been shown superior over qualitative evaluations and ^15^O-water PET is considered the gold standard for non-invasive quantification of MBF.^1^–^6^ For accurate estimation of tracer uptake, essential for quantification, a proper attenuation correction is required. PET/CT scanners make use of a CT scan directly before or after the PET scan to perform this correction. Misalignment between PET and CT acquisitions can induce artifacts in PET images and has been reported to be a common problem occurring in 50%-67% of cases.^7^,^8^ Misalignment can be induced by respiratory and patient body motion. CT scans are typically short and represent a ‘snap-shot’ of the respiratory cycle whereas the PET scan is averaged over many cycles, potentially leading to a misalignment, mainly in the cranio-caudal direction.^9^ In one study, an average motion of the heart of 8 mm during the PET acquisition was attributed directly to respiratory motion.^10^ in addition, patient body motion ranging between 5 and 10 mm commonly occurs.^11^

These motions frequently lead to a misalignment between PET and CT and induce errors in attenuation correction. In a qualitative analysis using ^82^Rb, false positive perfusion defects occurred and were induced by superimposed lung tissue on the anterior and lateral myocardial wall.^8^,^12^ In quantitative analysis of ^82^Rb, MBF was also shown to be mostly affected in the same regions but the effect extended over the whole myocardium.^13^

For all PET tracers except ^15^O-water, MBF is determined based on the uptake rate of the tracer, representing transmural MBF (MBFt). With ^15^O-water, however, MBF is determined primarily based on its washout rate rather than its uptake rate, representing MBF in perfusable tissue only, although ^15^O-water uptake rate resembles transmural MBF similarly as for the other tracers.^14^ In scarred regions, quantification of both MBFt and MBF when using ^15^O-water, adds information on scar burden in addition to ischemic burden in the perfusable and viable tissue. Since errors in attenuation correction due to misalignment affect the amplitude, but not the shape, of tissue time-activity curves, we hypothesize that misalignment may have less effects on washout-based MBF estimates for ^15^O-water than uptake-based MBFt that is used for other tracers.

The impact of misalignment on the quantitative accuracy of cardiac ^15^O-water PET has not, to the best of our knowledge, been evaluated yet in detail. Hence, the purpose of the present study was to investigate the influence of misalignment between PET and CT on the quantitative accuracy of washout-based MBF and uptake-based MBFt using ^15^O-water PET/CT.

## Materials and methods

### Patients

Stress data from 10 patients referred for routine clinical assessment of MBF with ^15^O-water PET was used in the present work. Misalignment between PET and CT was ruled out by visual inspection (excluding 4 out of 14) and no obvious dynamic patient motion during the PET scan could be seen. Since only anonymized images were used and the present work was purely an image processing study, this study did not require ethics permission according to the Swedish Law on Medical Research in Humans.

### Data acquisition

The protocol started with a low dose CT during normal breathing (120 kV, 10-20 mA, noise index 170, rotation time 1 second, pitch .53, scan duration 11.5 second, axial FOV 40 mm). Then, dynamic 4 minutes rest and stress PET/CT acquisitions were performed on a GE discovery MI (GE Healthcare, Waukesha, WI) using an automatic injection of 400 MBq ^15^O-water as a fast bolus (5 mL at 1 mL/s, followed by 35 mL saline at 2 mL/s). To induce hyperemic stress adenosine was infused (140 μg/kgmin) for 6 min starting 2 min prior to the PET/CT scan. Data were reconstructed into 20 frames (1 × 10, 8 × 5, 4 × 10, 2 × 15, 3 × 20, 2 × 30 seconds), using time-of-flight ordered subsets expectation maximization (TF-OSEM, 3 iterations and 16 subsets), including resolution recovery.

### Misalignment

Using ACQC (GE Healthcare, Waukesha), 11 different misalignments between PET and CT were induced in six different directions with 10 and 20 mm amplitudes using a right-handed coordinate system with PET shifted with respect to CT: caudal (+*Z*, 10 and 20 mm), cranial (− *Z*, 10 and 20 mm), lateral (± *X*, 10 and 20 mm), anterior (+*Y*, 10 mm), and anterior combined with cranial (10 mm + *Y*and 10 mm − *Z*, 10 mm + *Y*and 20 mm − *Z*). The combination of anterior and cranial misalignment (*Y*–*Z*) simulates a reaction induced by the stress agent, which can induce patient discomfort and altered posture with subsequent displacements in anterior and cranial direction from the original position. Misalignment was applied to all frames in the entire dynamic PET series.

### Quantification

The original scan with correct PET and CT alignment as well as all scans with an induced misalignment were analyzed in aQuant software (Medtrace Pharma A/S, Lyngby, Denmark). In short, a basis function implementation of the single-tissue compartment model with partial volume and spill over correction was used for creation of parametric MBF and perfusable tissue fraction (PTF) images.^15^–^18^

1 $$ C_{\text{PET}} \left( t \right) = {\text{MBF}} \times {\text{PTF}} \times C_{\text{A}} \left( t \right) \otimes e^{{ - \frac{\text{MBF}}{{V_{\text{D}} }}t}} + V_{\text{A}} C_{\text{A}} \left( t \right) + V_{\text{RV}} C_{\text{RV}} \left( t \right). $$

Here, *C*_A_(*t*) is the arterial blood radioactivity concentration, *C*_RV_ is the radioactivity concentration in the right ventricular cavity, *V*_A_ and *V*_RV_ are blood volume and spill-over factors and *V*_D_, the distribution volume of water, was fixed to .91 mL/g.^19^ As can be seen, MBF is present in both the washout and uptake rates and represents MBF in perfusable tissue only. Multiplication of MBF with PTF (i.e. the uptake rate) then results in total transmural MBFt, similar to other tracers. To correct for partial blood volume, MBFt was divided by 1 − *V*_A_. Segmentation of the myocardial wall was performed on PTF images, and MBF and MBFt were calculated on the global level and for the three coronary artery territories. To compare misalignment-induced changes in MBF to inter- and intra-observer variability the original scans were analyzed by two observers, of whom one analyzed the data twice.

### Statistics

Data are presented as mean ± standard deviation (SD). To analyze differences between misaligned and original scans, Bland–Altman analysis, Wilcoxon signed-rank test, and intra class correlation coefficient (ICC) for agreement was used. Observer variability was assessed using mean bias ± SD and ICC. A two-sided *P* value < .05 was considered significant. Statistical analysis was performed in Matlab (The Mathworks, Natick, Massachusetts).

## Results

**Figure 1:**
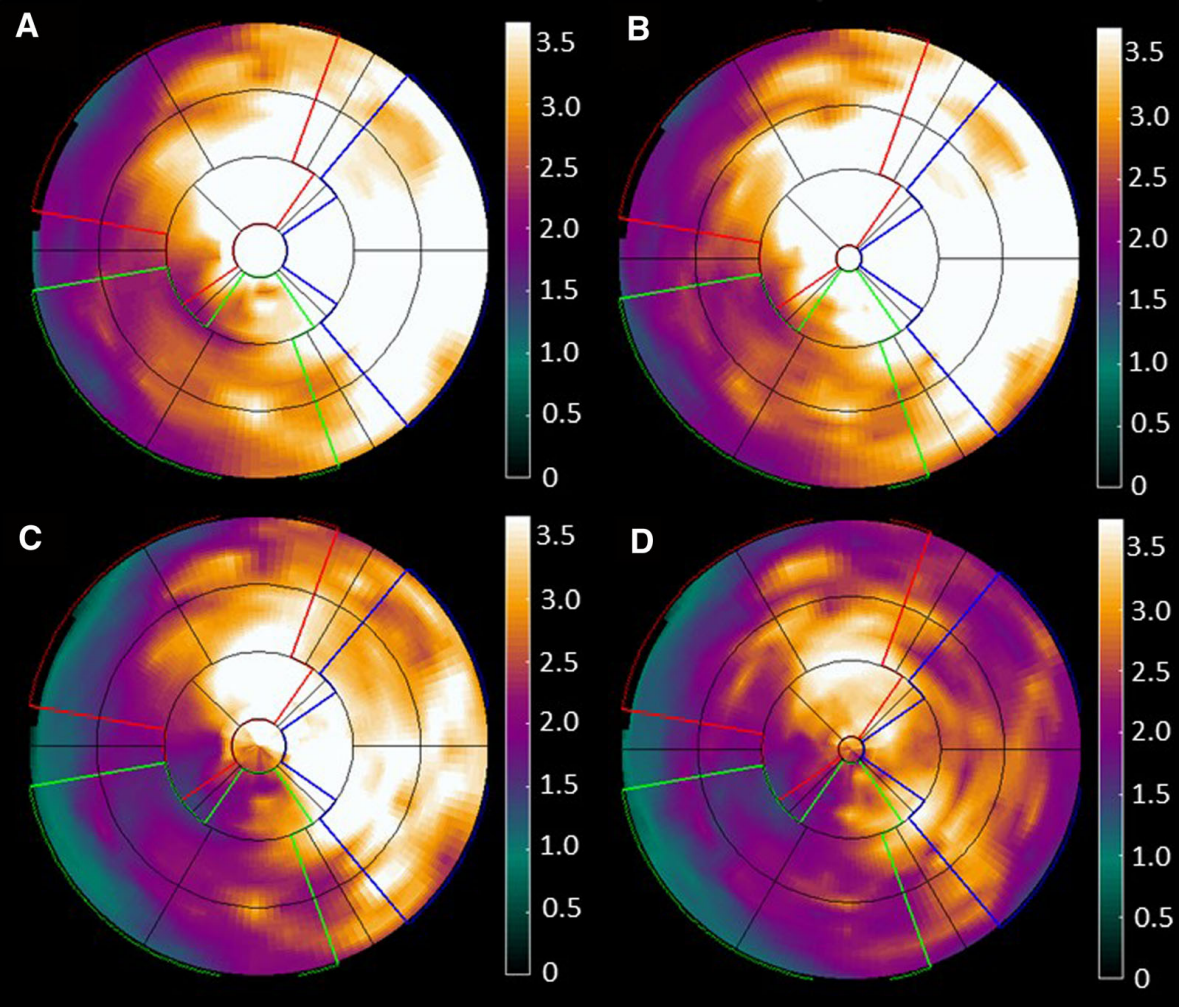
Polar plots of a patient showing only minor effects on myocardial blood flow (MBF) by misalignment *X* + 20 in **B** compared to the original scan without misalignment in **A**. On the other hand in **D**, transmural MBF is clearly affected in the left circumflex territory (LCX) with a decrease of 23% compared to the original scan in **C**

**Figure 2:**
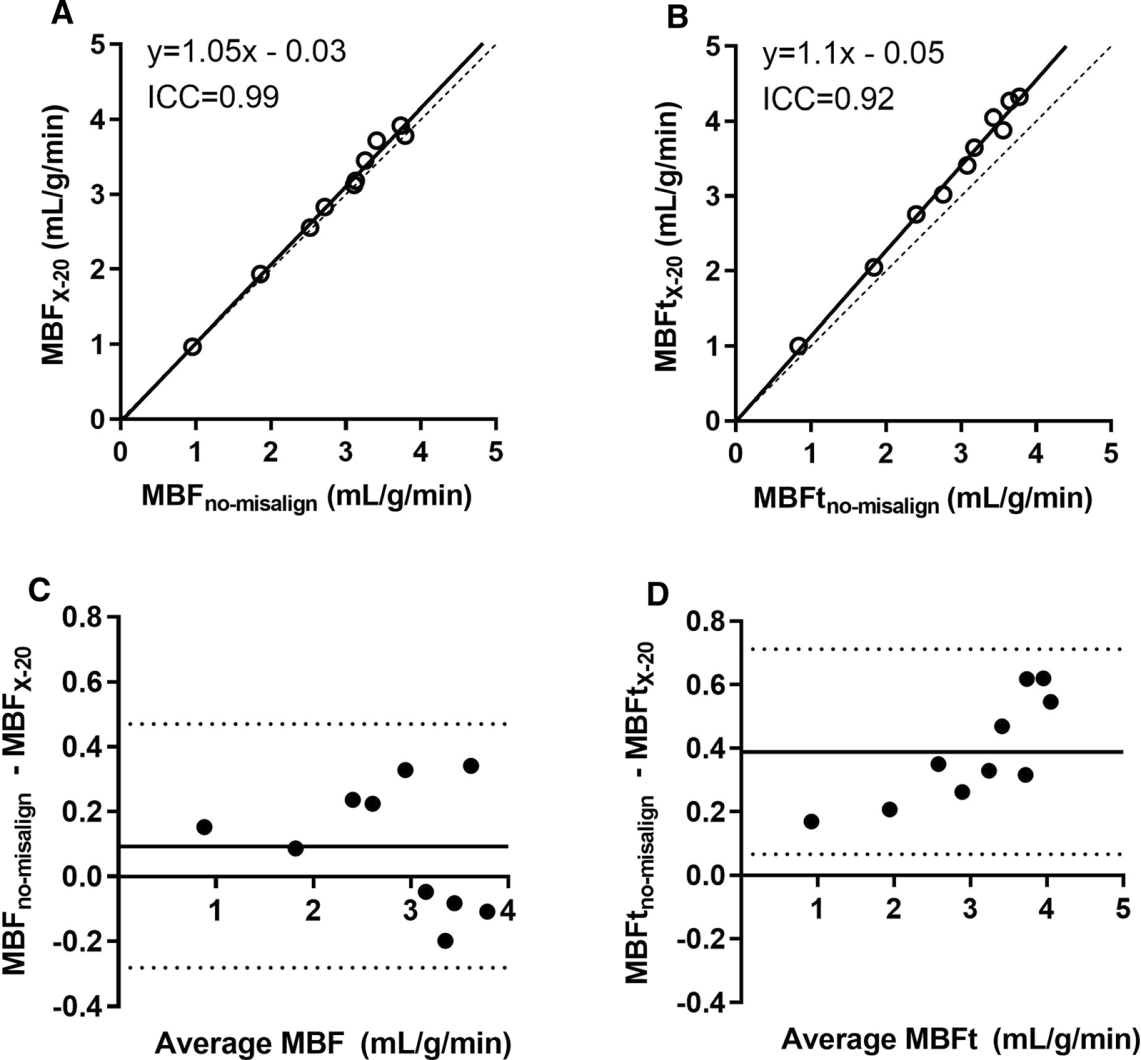
Linear regression and intra class correlation (ICC) in **A**, **B**, and Bland–Altman analysis in C-D of myocardial blood flow (MBF) and transmural MBF (MBFt) in the left circumflex territory (LCX) for 20 mm misalignment in the right lateral direction (*X* − 20). The solid lines in A-B are line of best fits and dashed lines are line of identity. In C-D the solid lines are mean bias and dashed lines are limits of agreement

**Figure 3:**
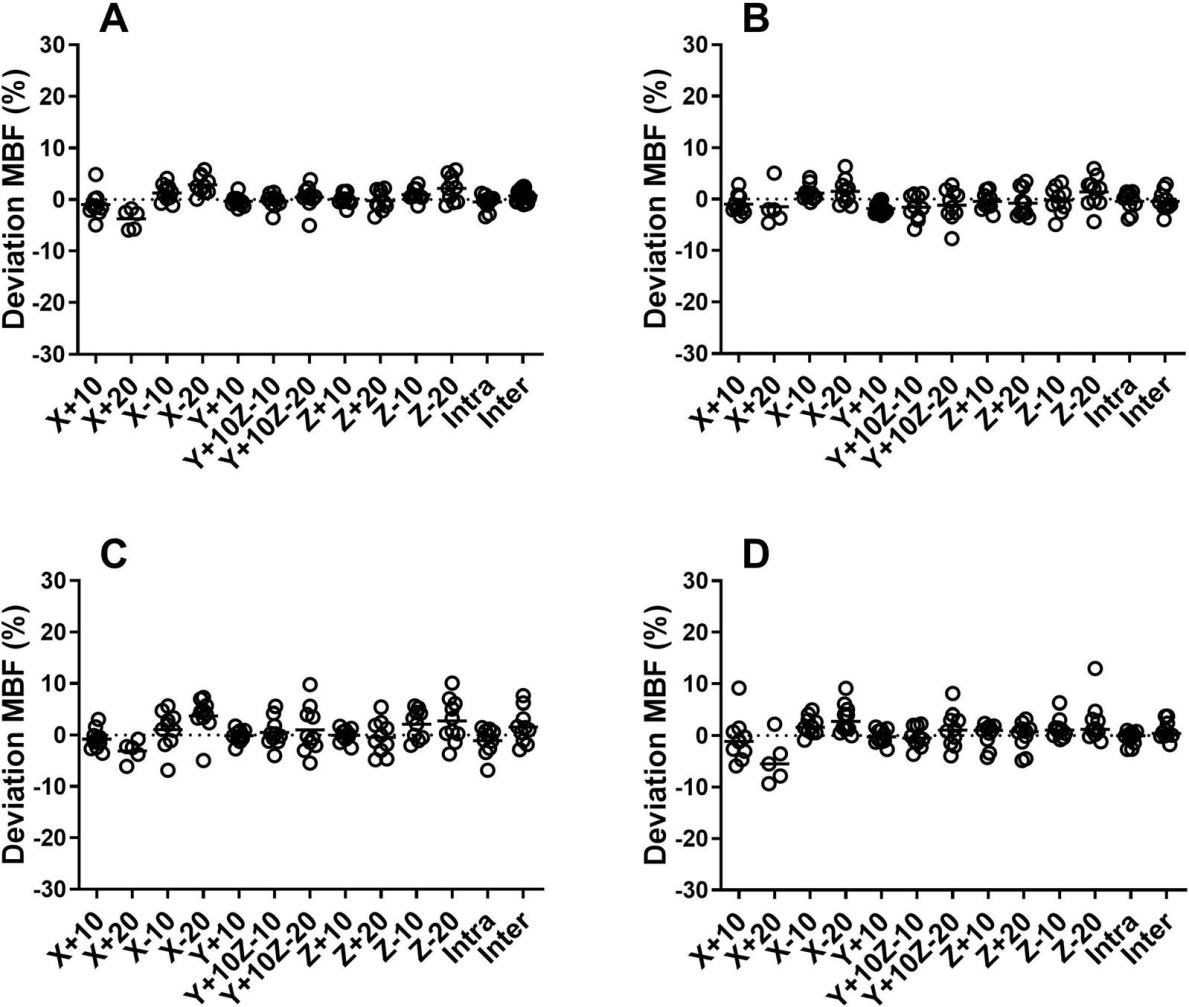
Scatter dot plots showing relative deviation of myocardial blood flow (MBF) in all misalignments compared to the original scan without misalignment for left ventricle (**A**), left anterior descending (**B**), right coronary artery (**C**), and left circumflex (**D**). Intra, intra-observer variability; Inter, inter-observer variability

**Figure 4:**
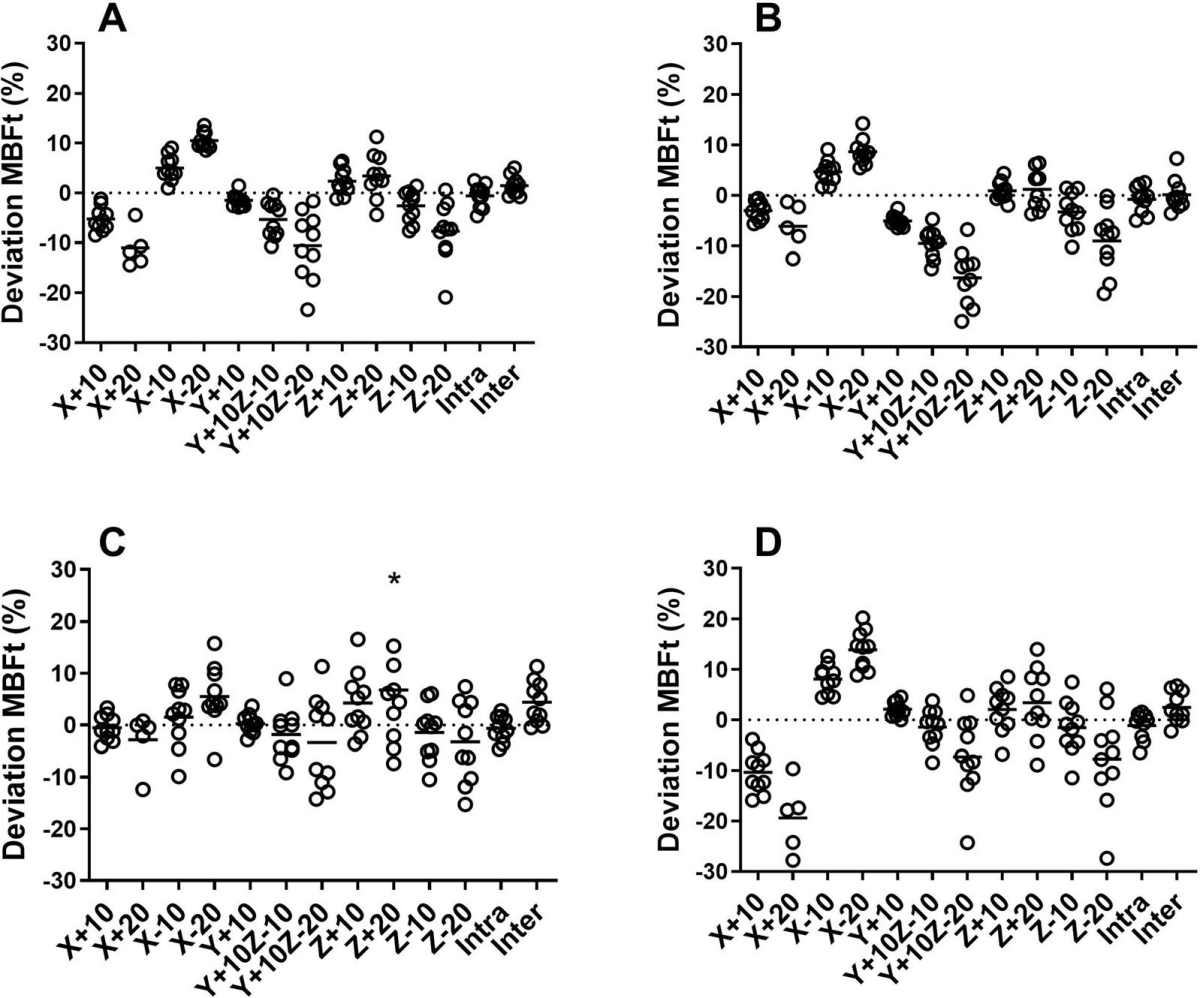
Scatter dot plots showing relative deviation of transmural myocardial blood flow (MBFt) in all misalignments compared to the original scan without misalignment for left ventricle (**A**), left anterior descending (**B**), right coronary artery (**C**), and left circumflex (**D**). Intra, intra-observer variability; Inter, inter-observer variability. * Deviation of 35.3% at one point in *Z* − 20

Global stress MBF ranged between 1.0 and 4.2 mL/g/min for all patients. For 5 patients, the results from 20 mm left-lateral misalignment (*X* + 20) were excluded since the resulting artifacts in PTF made the myocardial wall impossible to segment. Washout-based MBF was only to a minor extent affected by misalignments but larger effects were seen in uptake-based MBFt. In any of the three coronary regions, 5 misalignments showed a significant difference for MBF and 9 misalignments for MBFt. On the global level and across all misalignments, average absolute deviation for MBF was 1.7% ± 1.4% and for MBFt 5.4% ± 3.2%. ICC between misaligned and original images were excellent for MBF (≥ .98) and good for MBFt (≥ .87). Largest deviation for MBF was − 4.8% ± 5.8% (LCX, *X* + 20) and for MBFt − 19.3% ± 9.6% (LCX, *X* + 20). In Figure 1, polar plots are showing the effect of 20 mm left-lateral misalignment (*X* + 20) on MBF and MBFt for one patient. Linear regression and Bland–Altman analysis of one of the worst misalignments, 20 mm right-lateral misalignment (*X* − 20), are shown in Figure 2. Figures 3, 4 are scatter dot plots showing the relative deviations from the values based on correctly aligned PET and CT for MBF and MBFt, and corresponding relative deviations and ICC are shown in Tables 1, 2. Inter- and intra-observer agreement were excellent with ICC ≥ .95 for all parameters (Table 3).

**Table 1:** Relative deviation and ICC of MBF for each misalignment compared to the original scan without misalignment

Misalignment	LV		LAD		RCA		LCX	
	Bias (%)	ICC	Bias (%)	ICC	Bias (%)	ICC	Bias (%)	ICC
Washout-based MBF								
*X* + 10	− 1.0 ± 2.5	.99	− 1.0 ± 2.0	1.0	− .8 ± 2.1	1.0	− .7 ± 4.8	.99
*X* − 10	1.3 ± 1.6	1.0	1.2 ± 1.2*	1.0	1.1 ± 4.3	1.0	1.9 ± .9*	1.0
*Y* + 10	− .4 ± 1.3	1.0	− 1.8 ± 1.3**	1.0	− .1 ± 1.5	1.0	− .1 ± 1.7	1.0
*Z* + 10	1.0 ± 1.1*	1.0	− .2 ± 2.8	1.0	2.1 ± 2.8	1.0	1.4 ± 2.6*	1.0
*Z* − 10	.1 ± 1.4	1.0	− .4 ± 1.8	1.0	− .03 ± 1.5	1.0	.2 ± 2.9	1.0
*X* + 20	− 3.7 ± .5	.98	− 1.5 ± 4.7	.99	− 3.1 ± 1.8	.98	− 4.8 ± 5.8	.97
*X* − 20	2.9 ± 1.2**	.99	1.5 ± 2.1	1.0	3.7 ± 4.2*	.99	3.3 ± 1.9**	.99
*Z* + 20	2.2 ± 2.9*	.99	− 1.4 ± 3.2	.99	2.8 ± 3.6	.99	2.6 ± 5.0*	.98
*Z* − 20	− .2 ± 2.1	1.0	− .8 ± 2.7	1.0	− .4 ± 4.2	1.0	− .1 ± 3.3	1.0
*Y* + 10*Z* + 10	− .3 ± 1.7	1.0	− 1.6 ± 3.1	1.0	.5 ± 3.1	1.0	− .3 ± 2.4	1.0
*Y* + 10*Z* + 20	.5 ± 2.6	1.0	− 1.2 ± 3.9	1.0	1.0 ± 3.7	.99	1.2 ± 4.3	1.0

*MBF*, myocardial blood flow; *LV*, left ventricle; *LAD*, left anterior descending; *RCA*, right coronary artery; *LCX*, left circumflex. *P < .05 **P < .005

**Table 2:** Relative deviation and ICC of MBFt for each misalignment compared to the original scan without misalignment

Misalignment	LV		LAD		RCA		LCX	
	Bias (%)	ICC	Bias (%)	ICC	Bias (%)	ICC	Bias (%)	ICC
Uptake-based MBFt								
*X* + 10	− 5.3 ± 2.8**	.98	− 3.0 ± 1.9**	1.0	− .5 ± 2.8	1.0	− 10.3 ± 4.7**	.94
*X*− 10	5.0 ± 2.5**	.99	4.7 ± 1.4**	.99	− 1.5 ± 6.2	.99	8.1 ± 2.7**	.97
*Y* + 10	− 1.5 ± 1.6*	1.0	− 5.0 ± 1.2**	.99	.3 ± 1.4	1.0	2.9 ± 6.3**	1.0
*Z* + 10	− 2.6 ± 3.4*	.99	− 3.3 ± 3.8*	.99	− 1.4 ± 6.8	.98	− 1.5 ± 4.9	.98
*Z* − 10	2.4 ± 2.8*	.99	.9 ± 2.0	1.0	4.3 ± 7.3*	.97	2.1 ± 4.2	.99
*X* + 20	− 11.0 ± 5.6	.87	− 6.1 ± 6.2	.97	− 2.8 ± 6.9	.98	− 19.3 ± 9.6	.64
*X*− 20	10.5 ± 1.7**	.96	8.7 ± 1.7**	.97	5.5 ± 7.5*	.98	13.9 ± 3.0**	.92
*Z* + 20	− 7.8 ± 4.8**	.95	− 9.0 ± 5.2*	.95	− 3.2 ± 8.4	.96	− 7.7 ± 7.3	.91
*Z* − 20	3.5 ± 5.3	.98	1.2 ± 3.7	.99	6.8 ± 15.6	.90	3.4 ± 5.8	.97
*Y* + 10*Z* + 10	− 5.3 ± 3.4**	.98	− 9.5 ± 2.7**	.96	− 1.8 ± 6.2	.98	− 1.4 ± 2.8	.99
*Y* + 10*Z* + 20	− 10.6 ± 5.6**	.91	− 16.3 ± 4.5**	.87	− 3.6 ± 9.4	.95	− 7.3 ± 5.3*	.93

*MBFt*, transmural myocardial blood flow; *LV*, left ventricle; *LAD*, left anterior descending; *RCA*, right coronary artery; *LC*X, left circumflex. *P < .05 **P < .005

**Table 3:** Inter- and intra-observer variability

Mean bias (%)	LV	LAD	RCA	LCX
MBF inter-observer	.7 ± 1.1	.4 ± 2.0	1.6 ± 3.4	1.0 ± 1.8
MBF intra-observer MBFt inter-observer MBFt intra-observer	.5 ± 1.5 1.5 ± 1.9 .6 ± 2.3	.4 ± 1.9 .1 ± 3.1 .8 ± 2.7	1.0 ± 2.6 4.4 ± 4.1* .6 ± 2.4	.4 ± 1.4 2.5 ± 3.1* 1.1 ± 2.7

ICC	LV	LAD	RCA	LCX
MBF inter-observer	1.00	1.00	.99	1.00
MBF intra-observer MBFt inter-observer MBFt intra-observer	1.00 1.00 1.00	1.00 .99 1.00	1.00 .99 1.00	1.00 .99 1.00

*ICC*, intraclass correlation coefficient; *MBF*, myocardial blood flow; *MBFt*, transmural myocardial blood flow; *LV*, left ventricle; *LAD*, left anterior descending; *RCA*, right coronary artery; *LCX*, left circumflex. *P < .05 **P < .005

## Discussion

Misalignment between PET and CT is a common problem in cardiac PET. In this study the influence of misalignment on the quantitative accuracy of MBF measurements based on ^15^O-water PET was investigated. Using clinical ^15^O-water PET/CT data, misalignment at different amplitudes in the cranio-caudal, anterior, and lateral directions was simulated.

When using ^15^O-water, MBF is calculated from the washout rate which should not be affected by attenuation correction. Actually, quantification of MBF from ^15^O-water PET has been shown to be feasible even when attenuation (and scatter) correction was completely omitted.^20^ The results of the present study point in the same direction, with a small influence of attenuation correction on washout-based MBF estimates, even when attenuation correction is erroneously applied due to misalignment with the CT. Differences were significant in only 5 of the 11 tested misalignments and were generally small (average absolute error 1.7% ± 1.4%). In general, misalignment-induced biases were just slightly larger than the inter-observer biases, which were also considered small. However, it should be noted that even though deviations in MBF were small on average, inter-patient variation implies that misalignment could, in some cases, affect MBF on a consequential level with the largest deviation reaching 13% for one patient in this study (LCX, *Z* + 20). In addition, 20 mm left-lateral motion caused substantial PTF artifacts that made analysis impossible in 5 out of 10 patients, so no MBF values could be computed. Note however that 20 mm misalignment in left-lateral direction is an extreme case, hence it is likely to adversely affect each PET study irrespective of tracer used and can easily be identified during scan QC.

Not surprisingly, uptake-based MBFt was affected to a higher degree compared to MBF, with significant deviations in 9 of 11 misalignments. This is because MBFt is determined by the amplitude of the time activity curve which is highly affected by (erroneous) attenuation correction. Figures 3 and 4 also suggest a considerably larger inter-patient variability in the effects of misalignment for MBFt than for MBF. MBFt was more affected in LAD and LCX than in RCA, which is explained by the proximity of the anterior and lateral wall to lung tissue that has a large difference in attenuation compared to cardiac tissue. Consequentially, left-lateral misalignment (*X* +) showed the largest deviation of MBFt from the original scan. MBFt represents the uptake rate (*K*_1_) which is the parameter of use in quantification of MBF from all other flow tracers with a retention in the myocardium. In another study that simulated misalignment between PET and CT in ^82^Rb scans, MBF quantified from *K*_1_ was substantially underestimated (− 24% ± 15%) for 10 mm misalignment in both caudal and left-lateral direction (*X* + 10, *Z* − 10).^13^ This is much more than the worst case found in the present work, which was an underestimation of − 10.6% ± 4.9% in MBFt for misalignment of 20 mm left-lateral direction (*X* + 20). Note that misalignment results in errors in uptake rate *K*_1_ which are then further magnified when correcting for the limited extraction of ^82^Rb,^21^ which is the likely reason that the error for ^82^Rb was substantially larger than that of ^15^O-water based MBFt.

As mentioned above, for 20 mm left-lateral motion (*X* + 20), artefacts in PTF of the anterior and lateral wall were so severe that delineation was impossible in 50% of cases. PTF is used for segmentation of the images as it is more stable in the presence of obstructive coronary artery disease, resulting in un-biased delineation of the LV wall. Misalignment-induced errors in PTF could however contribute to uncertainties to the quantification of MBF in this particular misalignment type. The software uses the anatomical tissue fraction (ATF) to determine which voxels are inside the myocardial wall and for all voxels with ATF below a certain threshold, PTF is set to zero. For 20 mm left-lateral misalignment this apparently leads to complete deletion of the lateral wall. Data from all patients 20 mm left-lateral misalignment could be re-analyzed without ATF thresholding which resulted in a considerable variability, especially in RCA (MBF: .8% ± 7.4%, MBF_t_: − 1.0% ± 6.5%) and LCX (MBF: .2% ± 7.7%, MBF_t_: − 23.4% ± 9.9%) territories. Analyses without ATF thresholding could have been done for all misalignments but this generally complicates segmentation and should only be used as an alternative for severe misalignments.

In the clinical evaluation of ^15^O-water PET, 2.3 mL/g/min is used as an ischemic threshold for MBF.^3^ Utilizing this threshold on the coronary level, only one misalignment (20 mm left-lateral, *X* + 20) in one single patient induced a change in diagnosis with a false positive defect (.3% of total simulated regions). For MBFt, an ischemic threshold was determined at 1.8 mL/g/min for our work using linear regression with MBF based on data from the 10 original scans without misalignment. A change in diagnosis was induced by 8 misalignments for MBFt, resulting in a total of 9 false positive and 3 false negative regions (3.6%). This indicates that misalignments between PET and CT would be of less clinical importance for MBF compared to MBFt, but the number of patients is too low to draw any firm conclusions.

Misalignment between PET and CT originates to a large proportion from the different scanning durations of the two acquisitions. PET is performed during several minutes, giving an average position of the heart over many respiratory cycles. CT on the other hand represents a snap shot at a random point of the respiratory cycle, which typically does not match the average position from PET. Longer CT protocols (i.e. cine or low-pitch protocols), as used in the present work, result in a respiratory averaged position, which has been shown to decrease misalignment between PET and CT, at the cost of an increased radiation dose.^12^,^22^ For faster low dose protocols, end-expiration breath hold position should be utilized for minimization of misalignment.^23^ However, despite the use of optimized protocols, misalignment will still be frequent and image co-registration is justified. Several studies have proposed manual and automatic image co-registration methods for metabolically trapped tracers such as ^13^N-ammonia and ^82^Rb, clearly showing benefits of PET and CT image co-registration.^7^,^12^,^24^–^27^ So far, no image co-registration method between PET and CT has been proposed for ^15^O-water. However, since washout-based MBF calculated from ^15^O-water is less vulnerable to PET CT misalignments, as shown in this study, image co-registration is of less importance.

There are some limitations of the present study that should be noted. The number of included patients is small. However, the exact same directions and amplitudes of misalignment have been simulated in each patient and a higher number of patients would likely not alter the conclusion. Dynamic patient motion during the PET scan was not included in the simulation and the results apply only to in-between scan motion. The impact of misalignment on coronary flow reserve (CFR) could not be investigated since only stress scans were included. If misalignment is only present during stress, impact on CFR would be the same as for stress only. When only rest or both scans are affected it is not as straight-forward. However, stress scans are probably more prone to misalignment than rest due to the induced discomfort from the stress agent.

## Conclusion

Misalignment between PET and CT has no significant effect on the quantitative accuracy of MBF from ^15^O-water PET, in all but the most extreme cases. Washout-based estimates of MBF were accurate and within 1.7% ± 1.4% and were much more robust than uptake-based estimates considering their sensitivities to misalignments. This implies that it is likely that misalignment is less of a problem for ^15^O-water than for other perfusion tracers. When only MBF is of interest, correction for misalignment between ^15^O-water PET and CT images is not necessary.

## New knowledge gained

For assessment of ischemic burden using ^15^O-water PET and clearance-based MBF, image co-registration between PET and CT is not necessary.

## Electronic supplementary material

Below is the link to the electronic supplementary material.

Electronic supplementary material 1 (PPTX 472 kb)

Electronic supplementary material 2 (M4A 8017 kb)

## Abbreviations

MBF: Myocardial blood flow
MBFt: Transmural myocardial blood flow
PTF: Perfusable tissue fraction
PET: Positron emission tomography
CT: Computerized tomography
LAD: Left anterior descending artery
RCA: Right coronary artery
LCX: Left circumflex
LV: Left ventricle
ICC: Intra-class correlation coefficient

## References

[1] Dewey M, Siebes M, Kachelrieß M et al. Clinical quantitative cardiac imaging for the assessment of myocardial ischaemia. Nat Rev Cardiol 2020. 10.1038/s41569-020-0341-8 PMC7297668 32094693

[2] Danad I, Raijmakers PG, Driessen RS, Leipsic J, Raju R, Naoum C, Knuuti J, Maki M, Underwood RS, Min JK, Elmore K, Stuijfzand WJ, van Royen N, Tulevski II, Somsen AG, Huisman MC, van Lingen AA, Heymans MW, van de Ven PM, van Kuijk C, Lammertsma AA, van Rossum AC, Knaapen P (2017). Comparison of coronary CT angiography, SPECT, PET, and hybrid imaging for diagnosis of ischemic heart disease determined by fractional flow reserve. JAMA Cardiol..

[3] Danad I, Uusitalo V, Kero T, Saraste A, Raijmakers PG, Lammertsma AA, Heymans MW, Kajander SA, Pietila M, James S, Sorensen J, Knaapen P, Knuuti J (2014). Quantitative assessment of myocardial perfusion in the detection of significant coronary artery disease: Cutoff values and diagnostic accuracy of quantitative [(15)O]H2O PET imaging. J Am Coll Cardiol.

[4] Kajander S, Joutsiniemi E, Saraste M, Pietila M, Ukkonen H, Saraste A, Sipila HT, Teras M, Maki M, Airaksinen J, Hartiala J, Knuuti J (2010). Cardiac positron emission tomography/computed tomography imaging accurately detects anatomically and functionally significant coronary artery disease. Circulation.

[5] Kajander SA, Joutsiniemi E, Saraste M (2011). Clinical value of absolute quantification of myocardial perfusion with (15)O-water in coronary artery disease. Circ Cardiovasc Imaging.

[6] Stuijfzand WJ, Uusitalo V, Kero T et al. Relative flow reserve derived from quantitative perfusion imaging may not outperform stress myocardial blood flow for identification of hemodynamically significant coronary artery disease. Circ Cardiovasc Imaging 2015. 10.1161/CIRCIMAGING.114.002400 25596142

[7] Martinez-Möller A, Souvatzoglou M, Navab N (2007). Artifacts from misaligned CT in cardiac perfusion PET/CT studies: Frequency, effects, and potential solutions. J Nucl Med..

[8] Lautamäki R, Brown TL, Merrill J, Bengel FM (2008). CT-based attenuation correction in (82)Rb-myocardial perfusion PET-CT: Incidence of misalignment and effect on regional tracer distribution. Eur J Nucl Med Mol Imaging.

[9] Martinez-Möller A, Zikic D, Botnar RM (2007). Dual cardiac-respiratory gated PET: Implementation and results from a feasibility study. Eur J Nucl Med Mol Imaging..

[10] Koivumäki T, Nekolla SG, Fürst S (2014). An integrated bioimpedance: ECG gating technique for respiratory and cardiac motion compensation in cardiac PET. Phys Med Biol.

[11] Hunter CR, Klein R, Beanlands RS (2016). Patient motion effects on the quantification of regional myocardial blood flow with dynamic PET imaging. Med Phys.

[12] Gould KL, Pan T, Loghin C (2007). Frequent diagnostic errors in cardiac PET/CT due to misregistration of CT attenuation and emission PET images: A definitive analysis of causes, consequences, and corrections. J Nucl Med.

[13] Rajaram M, Tahari AK, Lee AH (2013). Cardiac PET/CT misregistration causes significant changes in estimated myocardial blood flow. J Nucl Med.

[14] Harms HJ, Lubberink M, de Haan S (2015). Use of a single 11C-meta-hydroxyephedrine scan for assessing flow-innervation mismatches in patients with ischemic cardiomyopathy. J Nucl Med.

[15] Watabe H, Jino H, Kawachi N, Teramoto N, Hayashi T, Ohta Y (2005). Parametric imaging of myocardial blood flow with 15O-water and PET using the basis function method. J Nucl Med.

[16] Boellaard R, Knaapen P, Rijbroek A, Luurtsema GJ, Lammertsma AA (2005). Evaluation of basis function and linear least squares methods for generating parametric blood flow images using 15O- water and Positron Emission Tomography. Mol Imaging Biol.

[17] Iida H, Rhodes CG, de Silva R, Yamamoto Y, Araujo LI, Maseri A (1991). Myocardial tissue fraction correction for partial volume effects and measure of tissue viabilityl. J Nucl Med.

[18] Hermansen F, Rosen SD, Fath-Ordoubadi F, Kooner JS, Clark JC, Camici PG (1998). Measurement of myocardial blood flow with oxygen-15 labelled water, comparison of different administration protocols. Eur J Nucl Med.

[19] Iida H, Kanno I, Takahashi A (1988). Measurement of absolute myocardial blood flow with H215O and dynamic positron-emission tomography Strategy for quantification in relation to the partial-volume effect. Circulation.

[20] Lubberink M, Harms HJ, Halbmeijer R (2010). Low-dose quantitative myocardial blood flow imaging using 15O-water and PET without attenuation correction. J Nucl Med.

[21] Moody JB, Murthy VL, Lee BC (2015). Variance estimation for myocardial blood flow by dynamic PET. IEEE TMI..

[22] Nye JA, Hamill J, Tudorascu D (2009). Comparison of low-pitch and respiratory-averaged CT protocols for attenuation correction of cardiac PET studies. Med Phys.

[23] Le Meunier L, Maass-Moreno R, Carrasquillo JA (2006). PET/CT imaging: Effect of respiratory motion on apparent myocardial uptake. J Nucl Cardiol..

[24] Slomka PJ, Diaz-Zamudio M, Dey D (2015). Automatic registration of misaligned CT attenuation correction maps in Rb-82 PET/CT improves detection of angiographically significant coronary artery disease. J Nucl Cardiol..

[25] Alessio AM, Kinahan PE, Champley KM (2010). Attenuation-emission alignment in cardiac PET/CT based on consistency conditions. Med Phys.

[26] Khurshid K, McGough RJ, Berger K (2008). Automated cardiac motion compensation in PET/CT for accurate reconstruction of PET myocardial perfusion images. Phys Med Biol.

[27] Marinelli M, Positano V, Tucci F et al. Automatic PET-CT image registration method based on mutual information and genetic algorithms. ScientificWorldJournal 2012. 10.1100/2012/567067 PMC3349214 22593696

