# Supplementary material for: Effect of PET-CT misalignment on the quantitative accuracy of cardiac 15O-water PET
Source: J Nucl Cardiol. 2020 Nov 4;29(3):1119–28. doi: 10.1007/s12350-020-02408-6 (PMC9163113; doi:10.1007/s12350-020-02408-6)
Supplement: Supplementary file 1 — Electronic supplementary material 1 (PPTX 472 kb) [file 12350_2020_2408_MOESM1_ESM.pptx]

## Slide 1
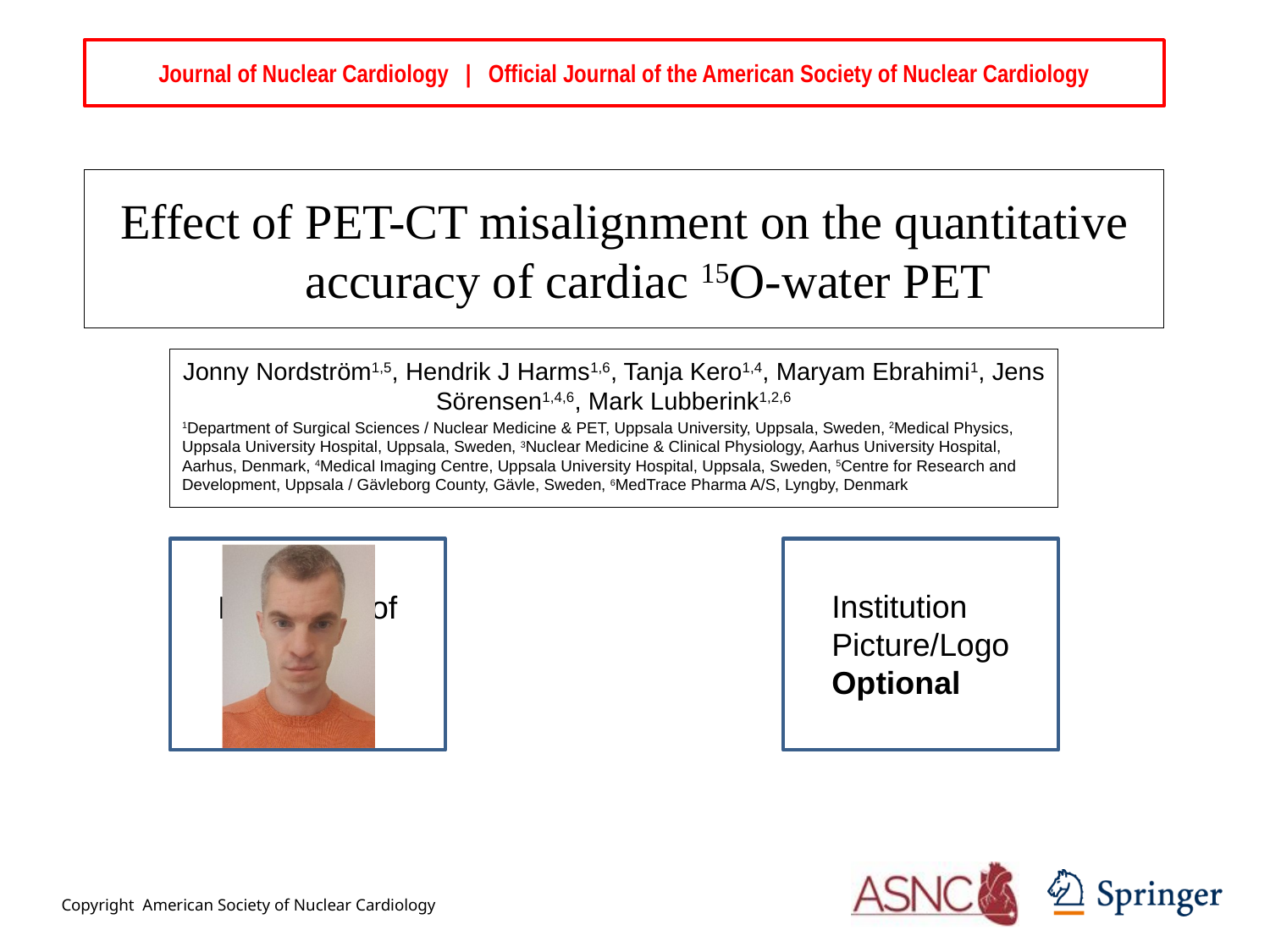

Journal of Nuclear Cardiology | Official Journal of the American Society of Nuclear Cardiology
# Effect of PET-CT misalignment on the quantitative accuracy of cardiac 15O-water PET
Jonny Nordström1,5, Hendrik J Harms1,6, Tanja Kero1,4, Maryam Ebrahimi1, Jens Sörensen1,4,6, Mark Lubberink1,2,6
1Department of Surgical Sciences / Nuclear Medicine & PET, Uppsala University, Uppsala, Sweden, 2Medical Physics, Uppsala University Hospital, Uppsala, Sweden, 3Nuclear Medicine & Clinical Physiology, Aarhus University Hospital, Aarhus, Denmark, 4Medical Imaging Centre, Uppsala University Hospital, Uppsala, Sweden, 5Centre for Research and Development, Uppsala / Gävleborg County, Gävle, Sweden, 6MedTrace Pharma A/S, Lyngby, Denmark
Head shot of author
required
Institution
Picture/Logo
Optional
Copyright American Society of Nuclear Cardiology

## Slide 2
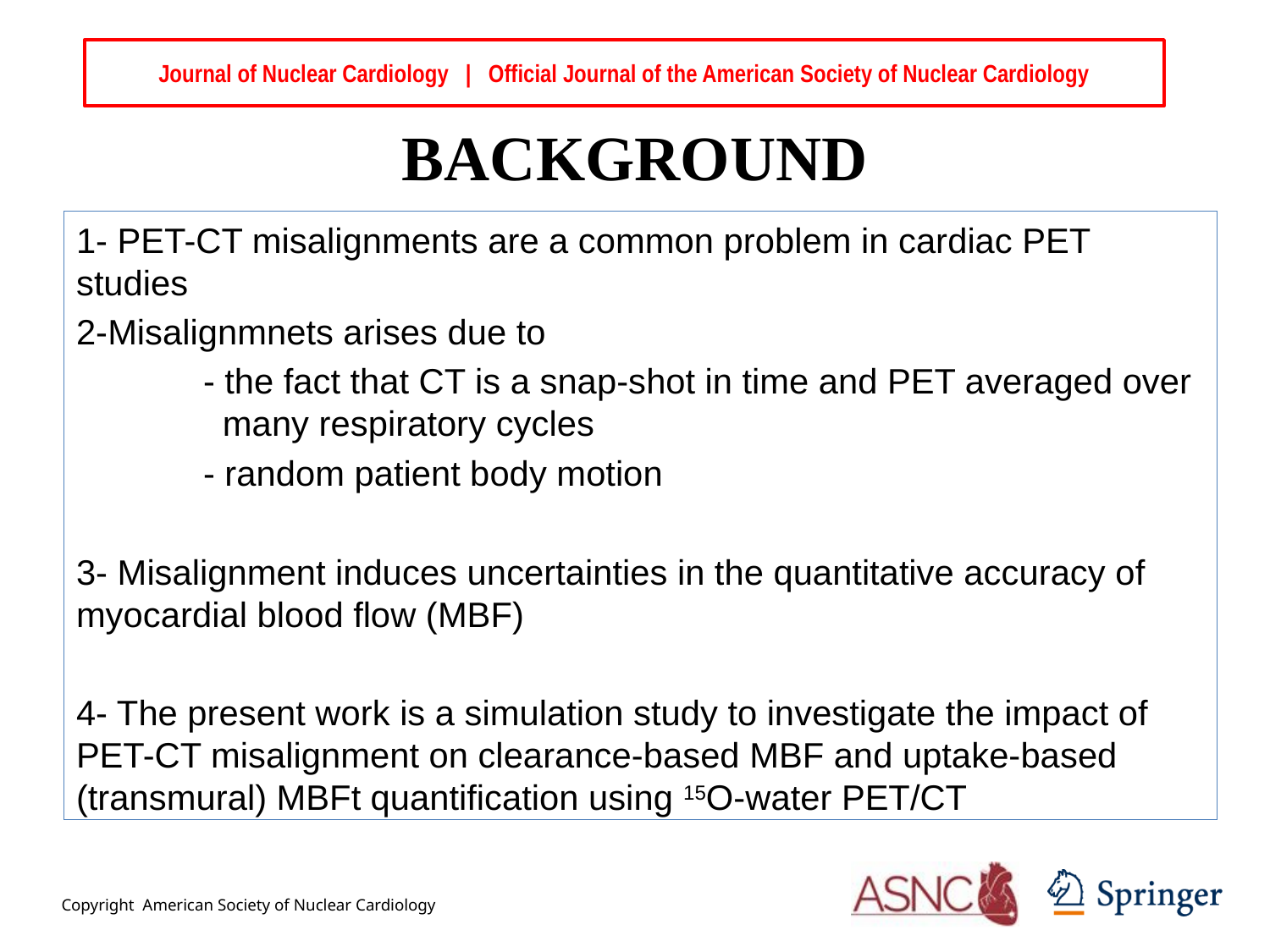

Journal of Nuclear Cardiology | Official Journal of the American Society of Nuclear Cardiology
# BACKGROUND
1- PET-CT misalignments are a common problem in cardiac PET studies
2-Misalignmnets arises due to
	- the fact that CT is a snap-shot in time and PET averaged over 	 many respiratory cycles
	- random patient body motion
3- Misalignment induces uncertainties in the quantitative accuracy of myocardial blood flow (MBF)
4- The present work is a simulation study to investigate the impact of PET-CT misalignment on clearance-based MBF and uptake-based (transmural) MBFt quantification using 15O-water PET/CT
Copyright American Society of Nuclear Cardiology

## Slide 3
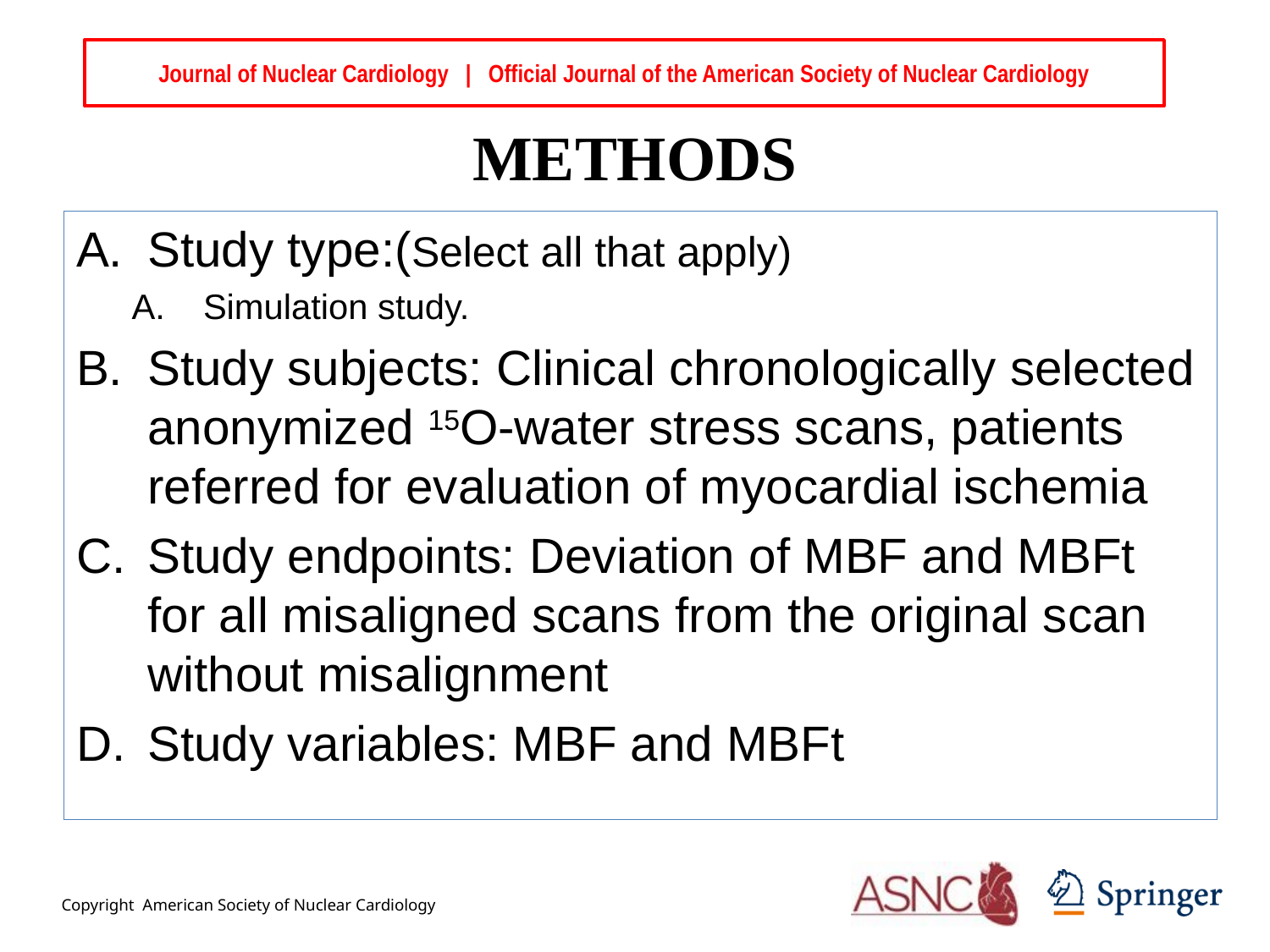

Journal of Nuclear Cardiology | Official Journal of the American Society of Nuclear Cardiology
# METHODS
Study type:(Select all that apply)
Simulation study.
Study subjects: Clinical chronologically selected anonymized 15O-water stress scans, patients referred for evaluation of myocardial ischemia
Study endpoints: Deviation of MBF and MBFt for all misaligned scans from the original scan without misalignment
Study variables: MBF and MBFt
Copyright American Society of Nuclear Cardiology

## Slide 4
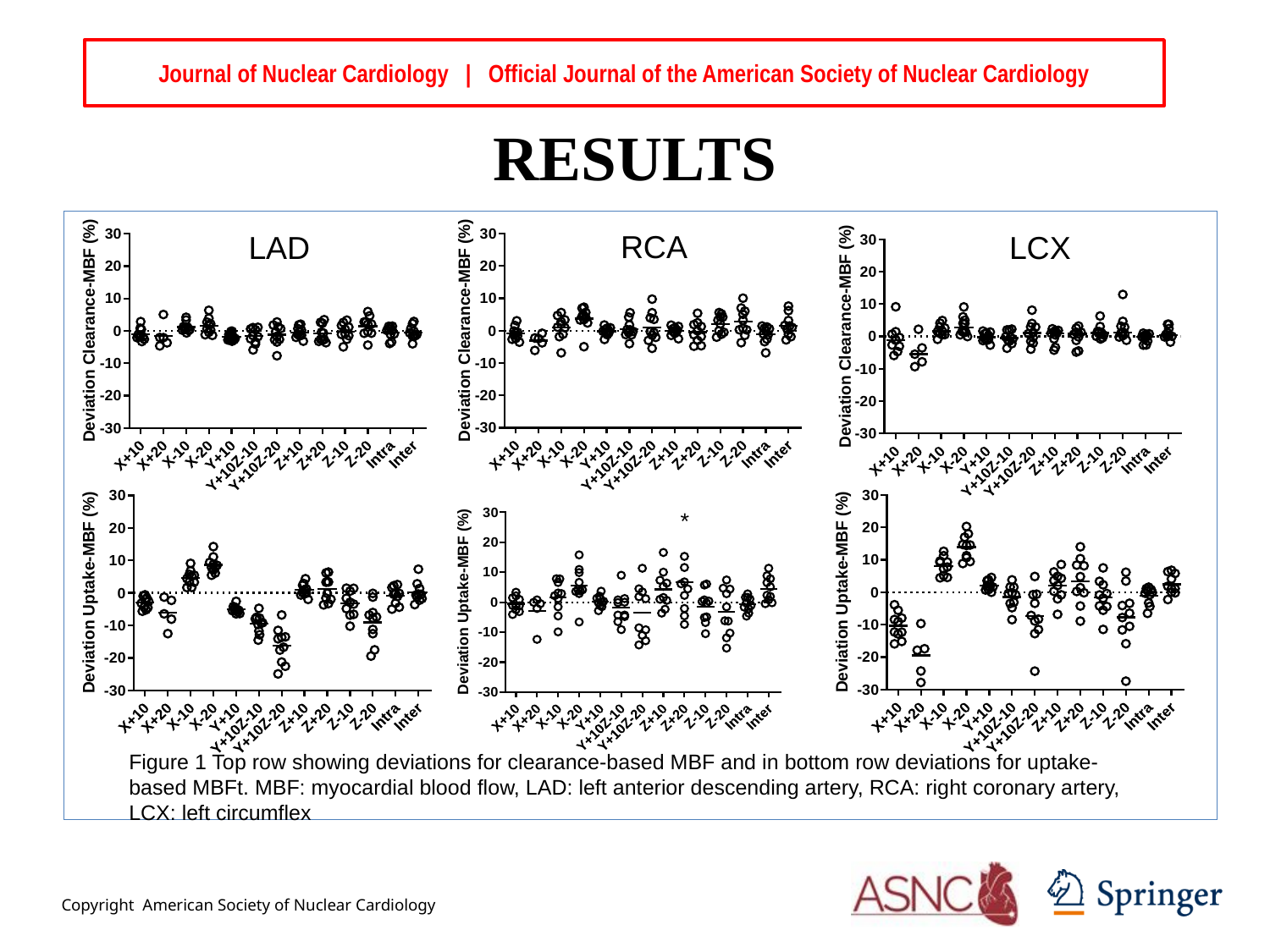

Journal of Nuclear Cardiology | Official Journal of the American Society of Nuclear Cardiology
# RESULTS
RCA
LAD
LCX
Figure 1 Top row showing deviations for clearance-based MBF and in bottom row deviations for uptake-based MBFt. MBF: myocardial blood flow, LAD: left anterior descending artery, RCA: right coronary artery, LCX: left circumflex
Copyright American Society of Nuclear Cardiology

## Slide 5
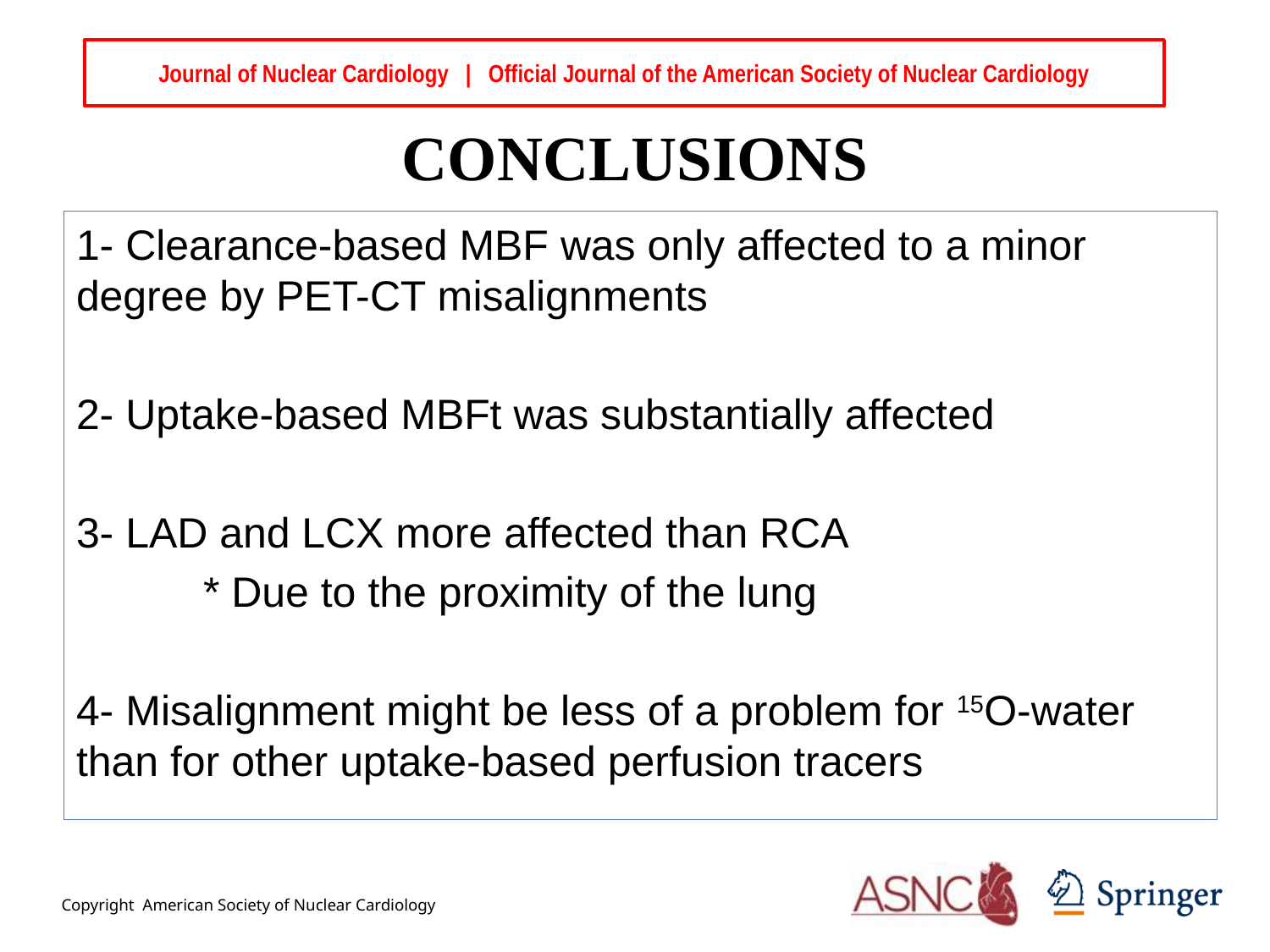

Journal of Nuclear Cardiology | Official Journal of the American Society of Nuclear Cardiology
# CONCLUSIONS
1- Clearance-based MBF was only affected to a minor degree by PET-CT misalignments
2- Uptake-based MBFt was substantially affected
3- LAD and LCX more affected than RCA
	* Due to the proximity of the lung
4- Misalignment might be less of a problem for 15O-water than for other uptake-based perfusion tracers
Copyright American Society of Nuclear Cardiology
